# Those are Your Legs: The Effect of Visuo-Spatial Viewpoint on Visuo-Tactile Integration and Body Ownership

**DOI:** 10.3389/fpsyg.2015.01749

**Published:** 2015-11-17

**Authors:** Polona Pozeg, Giulia Galli, Olaf Blanke

**Affiliations:** ^1^Center for Neuroprosthetics, School of Life Sciences, Ecole Polytechnique Fédérale de Lausanne, Lausanne, Switzerland; ^2^Laboratory of Cognitive Neuroscience, Brain Mind Institute, Ecole Polytechnique Fédérale de Lausanne, Lausanne, Switzerland; ^3^Istituti di Ricovero e Cura a Carattere Scientifico, Fondazione Santa Lucia, Rome, Italy; ^4^Department of Neurology, University Hospital of Geneva, Geneva, Switzerland

**Keywords:** crossmodal congruency effect, visuo-spatial viewpoint, body ownership, body illusion, legs

## Abstract

Experiencing a body part as one’s own, i.e., body ownership, depends on the integration of multisensory bodily signals (including visual, tactile, and proprioceptive information) with the visual top-down signals from peripersonal space. Although it has been shown that the visuo-spatial viewpoint from where the body is seen is an important visual top-down factor for body ownership, different studies have reported diverging results. Furthermore, the role of visuo-spatial viewpoint (sometime also called first-person perspective) has only been studied for hands or the whole body, but not for the lower limbs. We thus investigated whether and how leg visuo-tactile integration and leg ownership depended on the visuo-spatial viewpoint from which the legs were seen and the anatomical similarity of the visual leg stimuli. Using a virtual leg illusion, we tested the strength of visuo-tactile integration of leg stimuli using the crossmodal congruency effect (CCE) as well as the subjective sense of leg ownership (assessed by a questionnaire). Fifteen participants viewed virtual legs or non-corporeal control objects, presented either from their habitual first-person viewpoint or from a viewpoint that was rotated by 90°(third-person viewpoint), while applying visuo-tactile stroking between the participants legs and the virtual legs shown on a head-mounted display. The data show that the first-person visuo-spatial viewpoint significantly boosts the visuo-tactile integration as well as the sense of leg ownership. Moreover, the viewpoint-dependent increment of the visuo-tactile integration was only found in the conditions when participants viewed the virtual legs (absent for control objects). These results confirm the importance of first person visuo-spatial viewpoint for the integration of visuo-tactile stimuli and extend findings from the upper extremity and the trunk to visuo-tactile integration and ownership for the legs.

## Introduction

The experience of the body as one’s own (i.e., the sense of body ownership), and its location in space, critically depend on multisensory and sensorimotor integration of bodily signals (Gallagher, 2000; Jeannerod, 2003; Blanke and Metzinger, 2009; Longo et al., 2010; Tsakiris, 2010; Blanke, 2012). The multisensory representation of one’s body and its parts as well as body ownership are based on the integration and weighting of different sensory bodily inputs (proprioceptive, tactile, visual, auditory, vestibular, and visceral) according to spatio-temporal laws of multisensory bodily perception (Macaluso and Maravita, 2010; Blanke, 2012; Apps and Tsakiris, 2014; Samad et al., 2015). These bottom-up multisensory and motor signals are further integrated and compared with more stable, offline body representations, such as perceptual, conceptual and semantic knowledge of the body (Carruthers, 2008; de Vignemont, 2010; Longo et al., 2010; Serino and Haggard, 2010), as well as visual top-down signals about the form and position of the body (Blanke et al., 2015).

The sense of body ownership may fail in various neurological conditions producing erroneous and disturbed body perceptions, for example disownership of one’s hand in somatoparaphrenia (Vallar and Ronchi, 2009; Romano et al., 2014), ownership for supernumerary limbs (Halligan et al., 1993; Guterstam et al., 2011) or seeing one’s body from a third-person viewpoint as in out-of-body experiences (Blanke et al., 2002, 2004; Blanke and Arzy, 2005; Blanke and Mohr, 2005; Bunning and Blanke, 2005). Moreover, presenting conflicting multisensory information about the location and appearance of one’s body or body part can experimentally modify the sense of body ownership. For example, in the rubber hand illusion participants see a rubber hand while their real hand is hidden from view. When both, the rubber and real hands are stroked in synchrony, the visual information usually biases proprioceptive signals resulting in illusory ownership for the rubber hand and in the experience of illusory touch, that is the perception of feeling touch as arising from the rubber hand (Botvinick and Cohen, 1998). Successful induction of bodily illusion through multisensory conflicts have been shown at the level of fingers (Dieguez et al., 2009), hands (Rubber hand illusion: Botvinick and Cohen, 1998), feet (Lenggenhager et al., 2015) or an entire body (Full body illusion: Ehrsson, 2007; Lenggenhager et al., 2007).

It has been argued that in order to experience body ownership, this multisensory body representation needs to be coded in a common, egocentric reference frame, characterized by seeing the body from a first-person visuo-spatial viewpoint (Blanke et al., 2002; Petkova and Ehrsson, 2008; Blanke and Metzinger, 2009). Several studies have shown that illusory ownership over a rubber hand is strongest when the rubber hand is seen close to the body (Lloyd, 2007) and from a first-person viewpoint that is at an anatomically plausible position (Ehrsson et al., 2004; Tsakiris and Haggard, 2005; Costantini and Haggard, 2007; Guterstam et al., 2011). Conversely, an induction of the RHI was achieved also in the absence of a first-person viewpoint, when tactile stimulation of the rubber hand was only seen in a mirror (Bertamini et al., 2011). Similarly, seeing another person’s face (third-person viewpoint) being touched in synchrony with one’s own face, leads to an enfacement illusion, evoking changes in the mental representation of one’s own face (Tsakiris, 2008; Sforza et al., 2010; Tajadura-Jimenez et al., 2012). The effect of visuo-spatial viewpoint on whole body ownership has also been investigated by testing the effects of different viewpoints from which a body (Petkova et al., 2011; Pfeiffer et al., 2014) is seen. Several studies found that body ownership is stronger from first-person visuo-spatial viewpoints as compared to shifted or rotated viewpoints (Petkova and Ehrsson, 2008; Slater et al., 2010; Petkova et al., 2011; Maselli and Slater, 2013). Moreover, it has been shown that illusory ownership for a virtual body can also be induced when the virtual body is visually presented from a posterior third-person viewpoint (Lenggenhager et al., 2007; Aspell et al., 2009, 2013), but that additional third-person viewpoint changes did not modulate ownership for a virtual body (Pfeiffer et al., 2014). Also, the fact that in the evoked or spontaneous cases of out-of-body experiences people experience seeing their own body from a third-person point of view, despite reporting strong body ownership at the elevated and disembodied position (Blanke et al., 2002, 2004; Blanke and Mohr, 2005; Aspell et al., 2013), demonstrates that the relation between bodily self-consciousness (including body ownership) and the first-person visuo-spatial viewpoint is more complex than previously assumed.

Besides the visuo-spatial viewpoint, other factors have been shown to significantly affect the strength of bodily illusions. For example, ownership illusions decrease with longer delays between the visual and tactile stimulation and larger spatial separations of the visual and proprioceptive information about the hand location (Ehrsson et al., 2004; Costantini and Haggard, 2007; Lloyd, 2007), which is in line with temporal and spatial principles of multisensory integration (Holmes and Spence, 2005; Stein and Stanford, 2008). The illusion is also reduced with decreased anatomical resemblance of the stroked hand (normally in the form of different objects) with respect to the participants’ real hand (Haans et al., 2008; Tsakiris et al., 2010) or incongruent hand laterality (Tsakiris and Haggard, 2005), indicating the involvement of visual top-down modulation in the process of embodiment of the rubber hand.

The most commonly used measures to assess subjective experience of illusory ownership of body parts or of a whole body are questionnaire ratings (Botvinick and Cohen, 1998; Tsakiris and Haggard, 2005; Lenggenhager et al., 2007). Objective, reaction-time based evidence has been obtained by using the crossmodal congruency effect (CCE) task (Spence et al., 1998), in which participants are asked to respond to tactile stimuli applied to their body while ignoring visual distractor stimuli, which may occur at a congruent or an incongruent location with respect to the tactile (target) stimulus. The CCE has been previously used as an implicit measure of body ownership, showing that the CCE is associated with a self-attribution of an artificial hand (Pavani et al., 2000; Zopf et al., 2010) or virtual body (Aspell et al., 2009; Maselli and Slater, 2014).

Research on body ownership and bodily processing in general has mostly concentrated on hands (see Tsakiris, 2010, for a review) or body (see Blanke, 2012, for a review), and only few studies have investigated how the lower body is represented in the brain and whether this representation is different from the one of the hand (Schicke and Röder, 2006; Heed and Röder, 2008; Schicke et al., 2009; van Elk et al., 2013). In fact, there are numerous functional differences between upper and lower limbs, compatible with neural differences in body ownership mechanisms; in comparison to lower limbs, the hands are more frequently and with different complexity used for action and object manipulations; they have more degrees of freedom to move, can be positioned in a much wider portion of the peripersonal space, and frequently interact with other parts of the body. On contrary, the functional role of the legs mostly pertains to locomotion, and the range of their possible positions in space is smaller and is mostly restricted to the sagittal vertical plane of the body. As a consequence, the integration of multisensory stimuli related to the feet and surrounding space might differ from what is described for the hands (van Elk et al., 2013). While hand actions require hand-centered representation, locomotive actions require a representation that is foot-centered and centered on the body midline (Morton and Bastian, 2004; Kannape et al., 2010; Kannape and Blanke, 2013; Galli et al., 2015), suggesting that space and body are represented differently during manual and pedal actions. However, studies, which directly compared visuo-tactile integration of stimuli related to hands and feet, yielded inconclusive results. For example, it was shown that the multisensory representation of the feet does not differ from that of the hands as inferred from measures of multisensory integration such as the CCE or temporal order judgment tasks (Schicke and Röder, 2006; Schicke et al., 2009). However, another study confirmed that the magnitude of the CCE did not differ between hands and feet, but only when they were in an anatomical, uncrossed position, indicating a similar peripersonal space representation (van Elk et al., 2013). However, when the limbs were crossed, only hand CCEs were affected, but not feet CCEs, pointing to a difference between hands and feet in the integration of visual, tactile and proprioceptive signals.

In the present study we investigated whether visuo-tactile integration for leg stimuli (assessed by the CCE) and leg ownership depend on the visuo-spatial viewpoint and the anatomical similarity of the legs’ shape. In a virtual leg illusion, virtual legs were visually presented to the participants on a head-mounted display (HMD), so that they saw virtual legs as superimposed over their physical legs. The virtual legs were either shown from the participant’s habitual first-person viewpoint, or as rotated by 90°, to simulate a third-person viewpoint. To induce ownership for the virtual legs we followed the established protocol of visuo-tactile stimulation as for the rubber hand. Visuo-tactile stroking was applied either at a virtual legs or a virtual control object not resembling human legs (Pavani et al., 2000; Tsakiris and Haggard, 2005; Lenggenhager et al., 2007; Aspell et al., 2009, 2013; Salomon et al., 2012). We predicted that seeing legs in first-person viewpoint would result in stronger body ownership and higher CCE score as compared to the conditions where the legs are seen from the third-person viewpoint or where, instead of legs, wooden blocks are presented.

## Materials and Methods

### Participants

Nineteen right-handed healthy participants from the student population at Ecole Polytechnique Fédérale de Lausanne (EPFL) took part in the experiment (5 females, mean age 25.8 ± 3.8 years, range 18–33 years). All participants had normal or corrected-to normal sight and no psychiatric or neurological history. Their participation in the study was reimbursed (20 CHF). They had no previous experiences with the task or experimental paradigm. All participants gave written informed consent; the study was approved by the ethics committee of EPFL and was performed in accordance with the Declaration of Helsinki. The data of four participants were not included in the analysis due to a technical problem (two participants) and due to the below-chance performance at the CCE task (two participants).

### Virtual Leg Illusion Paradigm

The virtual leg illusion paradigm was adapted from the rubber hand illusion (Botvinick and Cohen, 1998) and full body illusion paradigms (Ehrsson, 2007; Lenggenhager et al., 2007; Petkova and Ehrsson, 2008) to study the role of visuo-spatial viewpoint in the embodiment of lower limbs. Subjects were comfortably sitting in a chair wearing a HMD (HMD, V-Real Viewer 3D SVGA, 800 × 600 pixels image resolution, 35° field of view, VRealities). The virtual legs or wooden objects were placed on another chair, mimicking a usual sitting position. A video camera recorded the virtual legs or wooden objects from a height and angle that corresponded to a subjective first person viewpoint and the video was projected in real time (except for asynchronous blocks, see below) onto the HMD. Thus, the subjects viewed the virtual legs or wooden objects as superimposed over their real legs (they were instructed to look in the direction of their legs). White noise was presented over headphones to mask the noise from the vibrators and surrounding. To induce the virtual leg illusion, the experimenter irregularly tapped (on average 2 taps per second) the participant’s left leg (dorsal surface between the knee and hip) and the corresponding virtual leg with a wooden stick. The subjects therefore viewed the virtual leg or wooden objects being tapped via the HMD and feel the touch applied to their real leg. Simultaneous tactile stimulation of the left and right leg would be preferred during illusion induction, however, due to the limitation of the manual application of tapping, only lateralized leg stimulation was possible.

**FIGURE 1:**
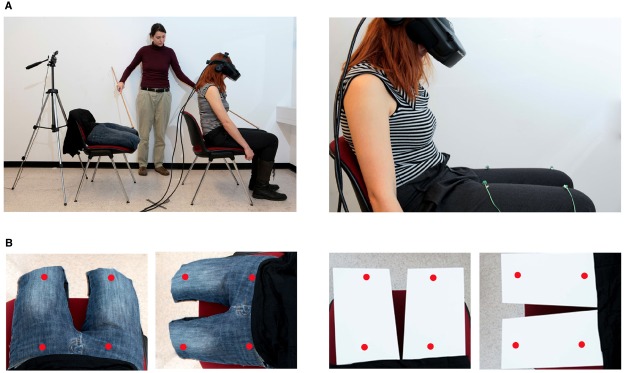
**Experimental setup. (A)** (LEFT) Virtual leg illusion. The experimenter simultaneously applies tactile stimuli to the participant’s leg and to the filmed virtual legs or wooden objects using a wooden stick. **(A)** RIGHT The participant wears a head-mounted display onto which the real-time (delayed in asynchronous condition) video of the virtual legs or wooden objects is projected. Four vibrating devices are attached to the participant’s legs. **(B)** (LEFT) The virtual legs or wooden objects as seen by the participant through the head-mounted display, presented in first- or third-person viewpoint. The red dots represent the positions of vibrotactile stimuli and visual distractors during the crossmodal congruency task. In the congruent conditions the vibrotactile stimulus and visual distractor appeared at the same location, whereas in the incongruent conditions they appeared at the same side but at a different position.

In the synchronous conditions the “seen tapping” and “felt tapping” matched spatially and temporally, whereas in the asynchronous blocks the visual information was delayed for 500 ms (using a video delaying device). The visuo-spatial viewpoint was manipulated by presenting subjects with the virtual legs (or wooden objects) in their habitual first-person viewpoint or in a third-person viewpoint, where the video image of the virtual legs (or wooden objects) was rotated by 90° anticlockwise. Each phase of illusion induction lasted for 60 s. The virtual leg illusion paradigm is shown in Figure 1.

### Crossmodal Congruency Effect Task

In order to study the role of visuo-spatial perspective in the embodiment of virtual legs or wooden objects, we adapted a behavioral task known as *Crossmodal distractor congruency task* by Spence et al. (1998). In this task participants are asked to make speeded judgments about the position of a tactile stimulus applied to their body, while ignoring visual distractor stimulus occurring at the spatially congruent or at an incongruent location with respect to the tactile (target) stimulus. The responses are usually slowed down when the distractor appears at an incongruent location. The reaction time (RT) difference between the incongruent and congruent trials is defined as CCE. The task has been extensively used to investigate multisensory integration in relation with peripersonal space and body representation as the CCE is larger when the visual distractors appear closer to the tactile (target) cue (Spence et al., 1998, 2004) or when the visual distractors are presented on a body part or whole body, which visually resemble a real human body (Pavani et al., 2000; Austen et al., 2004; Aspell et al., 2009). Thus, the task has been as such used as an implicit measure of hand (Pavani et al., 2000; Pavani and Castiello, 2004; Spence et al., 2004; Shore et al., 2006; Igarashi et al., 2010) and body ownership (Aspell et al., 2009; Maselli and Slater, 2014).

In the present study the vibrotactile stimuli were delivered by four vibration devices, each consisting of a small vibrating motor [Precision MicroDrives shaftless vibration motors, model 312–101, 3V, 60 mA, 9000 rpm (150 Hz), 5 g]. The motors had a surface area (the area touching the participant’s leg) of 113 mm^2^. The activation of the motors gave a clearly perceived and easy-to-localize stimulation. Two devices were attached to each of the participants’ legs using a medical tape: on each leg, the “upper” device was positioned approximately 3 cm from the knee and the “lower” device 25 cm below (Figure 1). The visual distractors were displayed through the HMD as a red dot at four different positions corresponding to the location of the vibrotactile motors. Vibro-tactile and visual distractors were presented simultaneously for 35 ms (stimuli onset was synchronous). In the congruent trials, the visual distractor appeared as superimposed to the activated vibrotactile stimulator (same position), whereas in the incongruent trials the visual distractor appeared at the opposite elevation, on the same side (same leg) according to the participant egocentric reference frame. The locations of appeared vibrotactile stimuli and visual distractors were balanced and randomized. The task in each experimental condition consisted of 96 trials (48 congruent and 48 incongruent; 48 were delivered with upper vibrotactile motors and 48 with lower; 48 on the right and 48 on the left leg). Stimulus timings were controlled by a program written with ExpyVR, a custom-built multimedia stimuli presentation software, developed with Python 2.6 and the Open Graphics Library v.2.2 (http://lnco.epfl.ch/).

The subjects were instructed to direct their gaze toward their real legs and fixate on the middle of the video conveyed through HMD. They were asked to make speeded judgments of whether they felt the vibrotactile stimulus at upper (knee level) or lower (hip level) position on their legs (with respect to their anatomical reference frame), regardless of the leg laterality, while ignoring the visual distractors. They responded by pressing one of two buttons on a keypad with their right hand. After the stimuli presentation, the participants had 2000 ms to respond to the tactile target with a button press before the next trial commenced.

### Virtual Leg Illusion Questionnaire

To assess the subjective experience of sense of ownership over the virtual legs or wooden objects, the participants were asked to rate 4 items from a questionnaire designed to capture the leg illusion, which was adapted from the rubber hand illusion (Botvinick and Cohen, 1998) and full body illusion (Ehrsson, 2007; Lenggenhager et al., 2007) questionnaires. The items referred to the sense of leg ownership, illusory touch, sense of motor agency over the displayed virtual legs or wooden objects, and illusion of proprioceptive leg rotation. We hypothesized that the sense of illusory ownership, illusory touch and illusion of motor agency would be stronger in the condition with synchronous visuo-tactile stimulation of the virtual legs shown in first-person viewpoint. On the other hand, the illusion of proprioceptive leg rotation should be stronger in the synchronous condition where the virtual legs are presented rotated, in the third-person viewpoint. The questionnaire items are presented in Table 1. Participants rated to which degree they agree with the item statement on a 7-item Likert scale ranging from –3 (“completely disagree”) to +3 (“completely agree”).

**TABLE 1:** **Virtual leg illusion questionnaire**.

	**Item**	**Label**
1	I had the impression that the legs/objects I was looking at were my real legs.	Ownership
2	I had the impression that the touch I saw was applied to my legs.	Illusory touch
3	I had the impression of being able to move the legs/objects.	Illusion of motor agency
4	I had the impression that my legs had changed position.	Illusion of proprioceptive rotation

The questionnaire included the four statements shown, describing illusory ownership, illusory touch, illusion of motor agency and illusion of proprioceptive leg rotation, which served as a control question for suggestibility. Participants indicated their response on a seven-point Likert scale ranging from “completely agree” (+++) to “completely disagree” (–––).

### Experimental Design

In a 2 × 2 × 2 repeated measures design we manipulated *Synchrony* of administered visuo-tactile tapping (Synchronous, Asynchronous), *Visuo-spatial viewpoint* (first-person, third-person), and *Body similarity* (virtual legs, wooden objects), thus in total encompassing 8 conditions. Each condition consisted of two illusion induction phases, where the experimenter applied visuo-tactile tapping to the participant’s leg and the virtual leg/wooden object for 60 s. Each illusion induction phase was followed by the CCE task (48 trials). The effects of the experimental manipulations on multisensory integration mechanisms were measured with the CCE scores on RT (see below) and error rate (ER). The subjective experience of embodiment was assessed with a questionnaire administered at the end of each condition.

### Procedure

The participants were first informed about the procedure of the experiment and asked to sign an informed consent to participate. While seated in a chair and wearing an HMD, the experimenter attached the vibro-tactile motors to the participant’s legs and aligned the video frame of the virtual legs or wooden objects to best fit the position of the vibro-tactile motors and the participant’s point of view. Then the participants were instructed about the CCE task and underwent a short training session, where the visual distractors were displayed on a black screen (16 trials). The experimental procedure was identical for all eight conditions. Subjects were instructed to orient their gaze in the direction of their legs, keep their eyes open and fixate on a location in the middle of the screen, as viewed via the HMD. Each condition consisted of two illusion-induction phases and two CCE task blocks (see the Experimental design section above). At the end of the condition the subjects removed the HMD and filled out the Illusion questionnaire. They were encouraged to take a short break before the subsequent block. The order of the conditions was randomized across the subjects.

### Data Analysis

The dependent measures of the experimental manipulations used for the data analyses were RT CCE scores, ER CCE and questionnaire ratings.

Crossmodal congruency effect scores were calculated by subtracting the mean RT (or ER) in congruent trials from the mean RT (or ER) in incongruent trials. Trials with incorrect responses and trials in which subjects failed to respond within 2000 ms were discarded from the RT analysis (on average, 2.3 % of trials), whereas the ER was calculated as the percentage of incorrect responses for all the valid trials (excluding only the trials with a failed response within 2000 ms). CCE scores were analyzed using repeated measures ANOVA with three within-subject factors: *Synchrony* (synchronous/asynchronous), *Visuo-spatial viewpoint* (first-person/third-person), and *Body similarity* (virtual legs/wooden objects). In the presentation and interpretation of RTs, we have mainly focused on the CCE scores from RT data rather than ER, as the RT CCE has been shown to be more sensitive (Pavani et al., 2000; Austen et al., 2004; Shore et al., 2006), however, we also report the ER in Table 2, and the ER CCE analyses.

**TABLE 2:** **Crossmodal congruency task results**.

	**Synchronous**		**Asynchronous**	
	**Congruent**	**Incongruent**	**Congruent**	**Incongruent**
***REACTION TIMES***				
**LEGS**				
1 POV	1220 (41)	1320 (49)	1200 (36)	1302 (43)
3 POV	1219 (42)	1234 (46)	1222 (36)	1246 (40)
**OBJECT**				
1 POV	1148 (40)	1215 (47)	1151 (40)	1216 (43)
3 POV	1170 (43)	1200 (41)	1166 (33)	1201 (40)
***ERROR RATES***				
**LEGS**				
1 POV	0.04 (0.01)	0.08 (0.02)	0.02 (0.01)	0.07 (0.01)
3 POV	0.04 (0.01)	0.04 (0.01)	0.03 (0.01)	0.06 (0.01)
**OBJECT**				
1 POV	0.03 (0.01)	0.04 (0.01)	0.01 (0.00)	0.05 (0.01)
3 POV	0.04 (0.01)	0.05 (0.02)	0.03 (0.01)	0.04 (0.01)

Average reaction times (in milliseconds, upper panel) and error rates (in percentages, lower panel) for virtual legs and wooden objects. Standard errors of the mean are shown in brackets. 1 POV, First-person viewpoint; 3 POV, Third-person viewpoint; LEGS, Virtual legs; OBJECT, Wooden objects.

Due to the ordinal type of the questionnaire data, the questionnaire scores were first ipsatized (Fischer and Milfont, 2010), and then analyzed with repeated measures ANOVA, using the same three within-subject factors as for the CCE task data. The significance (alpha) level used was 0.05. Significant interactions were followed up with planned pairwise comparisons using two-tailed paired *t*-tests. The alpha level of significance was adjusted accordingly to the number of comparisons using the Bonferroni method.

Pearson product-moment correlation coefficients were calculated to assess the relationship between the questionnaire ratings and CCE score.

## Results

### Crossmodal Congruency Effect. Reaction Times

**FIGURE 2:**
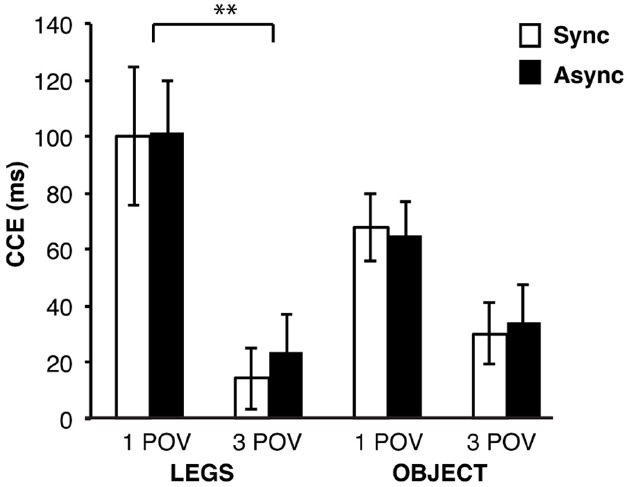
**CCE results.** The visuo-tactile integration was the strongest in the conditions where participants saw the virtual legs in the first-person viewpoint and significantly differed from the conditions when they saw the virtual legs in the third-person viewpoint, whereas the visuo-spatial viewpoint did not significantly affect the visuo-tactile integration when participants were seeing the wooden objects. The error bars depict the standard error of the mean. Sync, Synchronous; Async, Asynchronous; 1 POV, First-person viewpoint; 3 POV, Third-person viewpoint; LEGS, Virtual legs; OBJECT, Wooden objects. **p < 0.01.

The three-way repeated measures ANOVA on RT CCE showed a significant main effect of Visuo-spatial viewpoint [*F*(1,14) = 32.75, *p* < 0.001, ${\text{η}}_{\text{p}}^{\text{2}}$ = 0.70]: the CCE magnitude was larger when the viewed legs or control object were seen from the first-person viewpoint (*M* = 83.7 ms, SEM = 13.0 ms) than seen from the third-person viewpoint (*M* = 25.6 ms, SEM = 9.4 ms). Not significant were the main effects of Synchrony [*F*(1,14) = 0.25, *p* = 0.626, ${\text{η}}_{\text{p}}^{\text{2}}$ = 0.02] and Body similarity [*F*(1,14) = 1.70, *p* = 0.214, ${\text{η}}_{\text{p}}^{\text{2}}$ = 0.11]. Significant was the two-way interaction between the Visuo-spatial viewpoint and Body similarity [*F*(1,14) = 5.63, *p* = 0.033; ${\text{η}}_{\text{p}}^{\text{2}}$ = 0.29]. *Post hoc* analysis of this interaction showed a larger CCE in the first-person viewpoint as compared to the third-person viewpoint condition, but only when the participants viewed virtual legs [first-person viewpoint: *M* = 101.9 ms, SEM = 19.4 ms, third-person viewpoint: *M* = 19.2 ms, SEM = 10.6 ms; *t*(14) = 5.17, *p* < 0.001, α(corr) = 0.0125], and not when they viewed the control objects [first-person viewpoint: *M* = 66.5 ms, SEM = 9.2 ms, third-person viewpoint: *M* = 32.1 ms, SEM = 10.6 ms; *t*(14) = 2.76, *p* = 0.015, α(corr) = 0.0125]. The other two pairwise comparisons did not reach the level of significance after correction for multiple comparisons: no significant differences in the CCE were found between legs or objects when they were viewed in the first-person viewpoint [*t*(14) = 2.20, *p* = 0.044, α(corr) = 0.0125] or when they were viewed in the third-person viewpoint [*t*(14) = –1.34, *p* = 0.203, α(corr) = 0.0125]. Non-significant were the two-way interactions between Synchrony and Body similarity [*F*(1,14) = 0.17, *p* = 0.690, ${\text{η}}_{\text{p}}^{\text{2}}$ = 0.01] and between Synchrony and Visuo-spatial viewpoint [*F*(1,14) = 0.16, *p* = 0.695, ${\text{η}}_{\text{p}}^{\text{2}}$ = 0.01]. The three-way interaction was also not significant [*F*(1,14) < 0.01, *p* = 0.956, ${\text{η}}_{\text{p}}^{\text{2}}$ < 0.01]. The mean RTs for each condition are shown in Table 2. The CCE RTs are shown in Figure 2.

### Crossmodal Congruency Effect. Error Rates

The three-way repeated measures ANOVA on ER CCE scores showed a significant main effect of Visuo-spatial viewpoint [*F*(1,14) = 6.54, *p* = 0.023, ${\text{η}}_{\text{p}}^{\text{2}}$ = 0.32], where regardless of the synchrony of stroking, seeing the virtual legs or control objects in the first-person viewpoint (*M* = 0.3, SEM < 0.1) led to higher ER CCE scores than seeing them in the third-person viewpoint (*M* = 0.1, SEM < 0.1 ). Not significant were the main effects of Synchrony [*F*(1,14) = 2.33, *p* = 0.149. ${\text{η}}_{\text{p}}^{\text{2}}$ = 0.14] and Body similarity [*F*(1,14) = 1.53, *p* = 0.237, ${\text{η}}_{\text{p}}^{\text{2}}$ = 0.10]. Not significant were the two-way interactions between Synchrony and Visuo-spatial viewpoint [*F*(1,14) = 0.07, *p* = 0.790, ${\text{η}}_{\text{p}}^{\text{2}}$ = 0.01], between Synchrony and Body similarity [*F*(1,14) = 0.66, *p* = 0.429, ${\text{η}}_{\text{p}}^{\text{2}}$ = 0.05] and between Visuospatial viewpoint and Body similarity [*F*(1,14) = 1.96, *p* = 0.183, ${\text{η}}_{\text{p}}^{\text{2}}$ = 0.12]. The three-way interaction between all three experimental factors was also not significant [*F*(1,14) = 2.30, *p* = 0.152, ${\text{η}}_{\text{p}}^{\text{2}}$ = 0.14]. The mean ERs are shown in Table 2.

### Questionnaire Ratings

#### Ownership

The questionnaire item: “*I had the impression that the legs/objects I was looking at were my real legs*.” was used to assess the degree of body ownership over the displayed virtual legs/wooden objects. Data analysis of the item ratings revealed a significant main effect of Synchrony [*F*(1,14) = 6.39, *p* = 0.024, ${\text{η}}_{\text{p}}^{\text{2}}$ = 0.31], showing that illusory ownership was experienced more strongly when the visuo-tactile stimulation was synchronous (*M* = 0.2, SEM = 0.1), as compared to asynchronous (*M* = –0.2, SEM = 0.1). Significant were also the main effects of Visuo-spatial viewpoint [*F*(1,14) = 9.70, *p* = 0.008, ${\text{η}}_{\text{p}}^{\text{2}}$ = 0.41] and Body similarity [*F*(1,14) = 18.63, *p* = 0.001, ${\text{η}}_{\text{p}}^{\text{2}}$ = 0.57]. Thus, the sense of ownership was stronger when the legs/objects were seen from the first-person viewpoint (*M* = 0.3, SEM = 0.1), as compared to the third-person viewpoint (*M* = –0.3, SEM = 0.1), and when the participants saw the virtual legs (*M* = 0.6, SEM = 0.3) as compared to the control objects (*M* = –0.6, SEM = 0.3). Not significant were the two-way interaction effects between Synchrony and Visuo-spatial viewpoint [*F*(1,14) = 0.52, *p* = 0.482, ${\text{η}}_{\text{p}}^{\text{2}}$ = 0.04], between Synchrony and Body similarity [*F*(1,14) = 0.62, *p* = 0.446, ${\text{η}}_{\text{p}}^{\text{2}}$ = 0.04], and between Visuo-spatial viewpoint and Body similarity [*F*(1,14) = 3.14, *p* = 0.098, ${\text{η}}_{\text{p}}^{\text{2}}$ = 0.18]. The three-way interaction was also not significant [*F*(1,14) = 0.80, *p* = 0.387, ${\text{η}}_{\text{p}}^{\text{2}}$ = 0.05].

#### Illusory Touch

In order to assess the strength of experienced illusory touch during the visuo-tactile stimulation, the participants rated the questionnaire item: “*I had the impression that the touch I saw was applied to my legs.*” The data analysis showed significant main effects of Synchrony [*F*(1,14) = 23.39, *p* < 0.001, ${\text{η}}_{\text{p}}^{\text{2}}$ = 0.62], Visuo-spatial viewpoint [*F*(1,14) = 4.89, *p* = 0.044, ${\text{η}}_{\text{p}}^{\text{2}}$ = 0.26], and Body similarity [*F*(1,14) = 4.73, *p* = 0.047, ${\text{η}}_{\text{p}}^{\text{2}}$ = 0.25]. Thus, the experience of illusory touch was reported stronger when the visuo-tactile stimulation was synchronous (*M* = 1.0, SEM = 0.1), as compared to asynchronous (*M* = 0.2, SEM = 0.1). The intensity of the illusory touch was also larger when the legs or objects were presented in the participant’s first-person viewpoint (*M* = 0.5, SEM = 0.1), than when seen from the third-person viewpoint (*M* = 0.3, SEM = 0.1), and when the subjects saw the virtual legs (*M* = 0.6, SEM = 0.1) as compared to when they saw the objects (*M* = 0.2, SEM = 0.1). Not significant were the two-way interaction effects between Synchrony and Visuo-spatial viewpoint [*F*(1,14) = 0.51, *p* = 0.487, ${\text{η}}_{\text{p}}^{\text{2}}$ = 0.04], between Synchrony and Body similarity [*F*(1,14) = 0.15, p = 0.708, ${\text{η}}_{\text{p}}^{\text{2}}$ = 0.01], and between Visuo-spatial viewpoint and Body similarity [*F*(1,14) = 0.17, *p* = 0.688, ${\text{η}}_{\text{p}}^{\text{2}}$ = 0.01]. The interaction between the three experimental factors was also not significant [*F*(1,14) = 0.36, p = 0.559, ${\text{η}}_{\text{p}}^{\text{2}}$ = 0.03].

#### Illusion of Agency

As the sense of embodiment also comprises of the sense of being in control of a body or a body part, we asked participant to report the strength of experienced sense of motor agency by rating the questionnaire item: “*I had the impression of being able to move the virtual legs/objects*.” The analysis of the item ratings showed a significant main effect of Synchrony [synchronous: *M* = 0.0, SEM = 0.1; asynchronous: *M* = –0.4, SEM = 0.1; *F*(1,14) = 14.90, *p* = 0.002, ${\text{η}}_{\text{p}}^{\text{2}}$ = 0.52], Visuo-spatial viewpoint [first-person viewpoint: *M* = 0.0, SEM = 0.1; third-person viewpoint: *M* = –0.3, SEM = 0.1; *F*(1,14) = 6.17, *p* = 0.026, ${\text{η}}_{\text{p}}^{\text{2}}$ = 0.31] and Body similarity [legs: *M* = 0.1, SEM = 0.1; objects: –0.5, SEM = 0.1; *F*(1,14) = 13.52, *p* = 0.002, ${\text{η}}_{\text{p}}^{\text{2}}$ = 0.49]. Significant was the two-way interaction between Synchrony and Visuo-spatial viewpoint [*F*(1,14) = 6.84, *p* = 0.020, ${\text{η}}_{\text{p}}^{\text{2}}$ = 0.33], but not between Synchrony and Body similarity [*F*(1,14) = 0.01, *p* = 0.934, ${\text{η}}_{\text{p}}^{\text{2}}$ < 0.01] or between Body similarity and Visuo-spatial viewpoint [*F*(1,14) = 2.14, *p* = 0.165, ${\text{η}}_{\text{p}}^{\text{2}}$ = 0.13]. We have found a significant three-way interaction between Body similarity, Visuo-spatial viewpoint and Synchrony [*F*(1,14) = 6.10, *p* = 0.027, ${\text{η}}_{\text{p}}^{\text{2}}$ = 0.30]. The *post hoc* analysis of the interaction effect revealed only one significant pairwise comparison, showing that the sense of motor agency was stronger when the participants were seeing objects in the first-person viewpoint during synchronous visuo-tactile stimulation (*M* = –0.1, SEM = 0.2), as compared to seeing them during asynchronous stimulation [*M* = –0.8, SEM = 0.2; *t*(14) = 3.87, *p* = 0.002, α(corr) = 0.008], although the average ratings in both conditions were low. Other pairwise comparisons did not reach the level of significance after the correction for multiple comparisons, adjusted at α(corr) = α/6 = 0.008 [Legs-1POV-Sync/Legs-1POV-Async: *t*(14) = 1.78, *p* = 0.096; Legs-1POV-Sync/Legs-3POV-Sync: *t*(14) = 2.00, *p* = 0.066; Legs-1POV-Sync/Objects-1POV-Sync: *t*(14) = 2.79, *p* = 0.014; Legs-3POV-Sync/Objects-3POV-Sync: *t*(14) = 1.97, *p* = 0.069; Objects-1POV-Sync/Objects-3POV-Sync: *t*(14) = 2.04, *p* = 0.060].

#### Illusion of Proprioceptive Leg Rotation

In order to assess the subjective changes in proprioceptive sense of participant’s legs position due to the manipulation of viewpoint we used the questionnaire item: “*I had impression that my legs had changed position*.”

The analysis of the item ratings revealed that the main effect of Synchrony was not significant [synchronous: *M* = –0.2, SEM = 0.1; asynchronous: *M* = –0.3, SEM = 0.1; *F*(1,14) = 2.25, *p* = 0.156, ${\text{η}}_{\text{p}}^{\text{2}}$ = 0.14]. Significant where the main effects of Visuo-spatial viewpoint [first-person viewpoint: *M* = –0.5, SEM = 0.1; third-person viewpoint: *M* = 0.0, SEM = 0.2; *F*(1,14) = 5.19, *p* = 0.039, ${\text{η}}_{\text{p}}^{\text{2}}$ = 0.27] and Body similarity [legs: *M* = 0.2, SEM = 0.2; objects: *M* = –0.6, SEM = 0.1; *F*(1,14) = 11.34, *p* = 0.005, ${\text{η}}_{\text{p}}^{\text{2}}$ = 0.45]. We found a significant two-way interaction between Synchrony and Visuo-spatial viewpoint [*F*(1,14) = 17.88, *p* = 0.001, ${\text{η}}_{\text{p}}^{\text{2}}$ = 0.56]. Further *post hoc* analysis of the interaction effect showed that the proprioceptive illusion of leg rotation was stronger during synchronous visuo-tactile stimulation, but only when the legs or objects were presented in the third-person viewpoint [synchronous: *M* = 0.3, SEM = 0.2; asynchronous: *M* = –0.2, SEM = 0.2; *t*(14) = 3.71, *p* = 0.002, α(corr) = 0.0125] and not when seen in the first-person viewpoint [synchronous: *M* = –0.6, SEM = 0.1; asynchronous: *M* = –0.4; SEM = 0.1; *t*(14) = 1.01, *p* = 0.332, α(corr) = 0.0125]. Seeing the legs or objects in the third-person viewpoint during synchronous visuo-tactile stimulation also resulted in stronger proprioceptive illusion than seeing them in the first-person viewpoint [*t*(14) = 4.00, *p* = 0.001, α(corr) = 0.0125]. The differences in average ratings between first- and third-person viewpoint when legs or objects were seen during asynchronous stroking were not significant [*t*(14) = –0.79, *p* = 0.445]. Not significant were the two-way interactions between Synchrony and Body similarity [*F*(1,14) = 0.33, *p* = 0.575, ${\text{η}}_{\text{p}}^{\text{2}}$ = 0.02] and between Visuo-spatial viewpoint and Body similarity [*F*(1,14) = 0.82, *p* = 0.382, ${\text{η}}_{\text{p}}^{\text{2}}$ = 0.06]. Not significant was also the three-way interaction between the experimental factors [*F*(1,14) = 3.41, *p* = 0.086, ${\text{η}}_{\text{p}}^{\text{2}}$ = 0.20].

**FIGURE 3:**
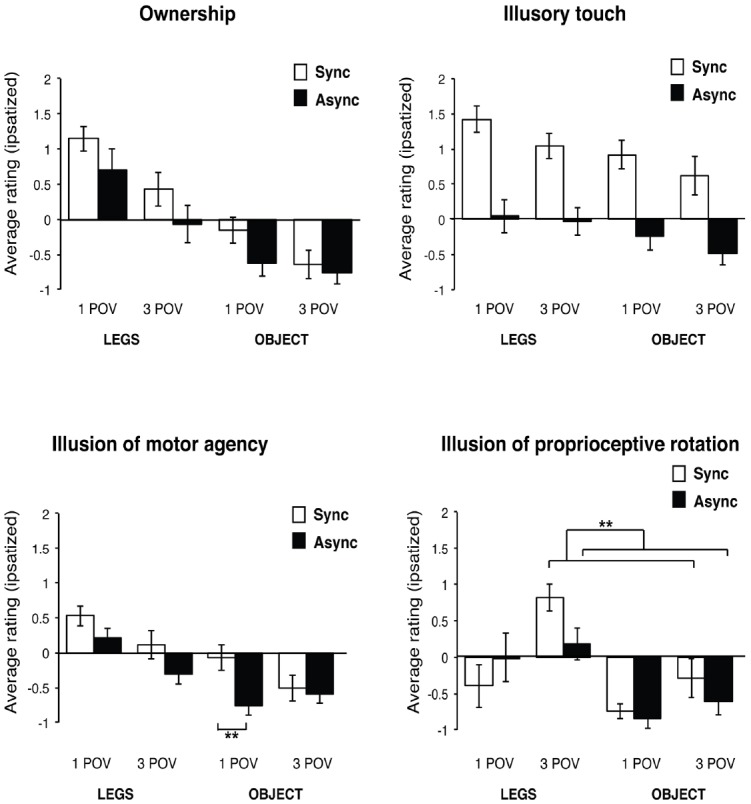
**Questionnaire results.** The figure represents the average ipsatized ratings of the Virtual leg illusion questionnaire. *Ownership*: The ratings of the experienced illusory ownership were higher when the participants were seeing the virtual legs (*Body similarity*: *p* = 0.001), when the dummy legs or objects were seen from the first-person viewpoint (*Visuo-spatial viewpoint*: *p* = 0.008) and when the visuo-tactile stimulation was synchronous (*Synchrony*: *p* = 0.024). *Illusory touch*: The strength of the experienced illusory touch was stronger in the conditions where participants saw the virtual legs (*Body similarity*: *p* = 0.047), when the legs or objects were presented in the first-person viewpoint (*Visuo-spatial viewpoint*: *p* = 0.044), and when the visuo-tactile stimulation was synchronous (*Synchrony*: *p* < 0.001). *Illusion of motor agency*: The sense of motor agency was the strongest during the condition where the virtual legs were seen in the first-person viewpoint and stimulated in synchrony with participants’ real legs (three-way interaction: *p* = 0.027). *Illusion of proprioceptive rotation*: The illusory sense that legs have changed position was rated the strongest in the conditions where the visuo-tactile stimulation of virtual legs or objects was synchronous and they were presented in the third-person viewpoint (*Visuo-spatial viewpoint* × *Synchrony*: *p* = 0.001). The error bars depict the standard error of the mean. Sync, Synchronous; Async, Asynchronous; 1 POV, First-person viewpoint; 3 POV, Third-person viewpoint; LEGS, Dummy legs; OBJECT, Wooden objects. **p < 0.01.

Based on the prediction that the illusion of proprioceptive leg rotation should be stronger in the synchronous condition where the virtual legs are presented in the third-person viewpoint as compared to the first-person viewpoint, and thus result in different pattern of responding across conditions as compared to the other three questionnaire items, the ratings of this item were used as a control for a bias in responding to the other three questionnaire items due to suggestibility or social desirability. Thus, we compared the ratings of this item (Illusion of proprioceptive leg rotation) in the condition where the virtual legs were viewed from the first-person viewpoint during synchronous visuo-spatial stimulation with the ratings of other three questionnaire items (Illusory ownership, Illusory touch and Illusion of motor agency) of the same condition. The ratings of the *Illusion of proprioceptive leg rotation* item were significantly lower than the ratings of the *Illusory ownership* [two-tailed paired *t*-test: *t*(14) = 5.83, *p* < 0.001, α(corr) = 0.017], *Illusory touch* [two-tailed paired *t*-test: *t*(14) = 5.94, *p* < 0.001, α(corr) = 0.017] and *Illusion of motor agency* [two-tailed paired *t*-test: *t*(14) = 3.00, *p* = 0.010, α(corr) = 0.017] of the same condition. The average ipsatized questionnaire ratings are shown in Figure 3.

### Correlation Between CCE and Questionnaire Data

Pearson product-moment correlation coefficients were calculated to assess the relationship between the questionnaire ratings and CCE score. In particular, we correlated the ratings of the four questionnaire items of the condition where participants viewed the virtual legs from a first-person viewpoint during synchronous visuo-tactile stimulation with the CCE score obtained during the same condition. None of the correlation coefficients was statistically significant [Illusory ownership: *r*(15) = 0.21, *p* = 0.450; Illusory touch: *r*(15) = 0.32, *p* = 0.238; Illusion of motor agency: *r*(15) = –0.05, *p* = 0.850; Illusion of proprioceptive leg rotation: *r*(15) = 0.36, *p* = 0.190].

## Discussion

The present study investigated how visuo-tactile integration and leg ownership depend on the visuo-spatial viewpoint using the virtual leg illusion paradigm. The participants were viewing either legs or control objects from either a first-person or third person viewpoint on an HMD, while receiving synchronous or asynchronous visuo-tactile stimulation. The study revealed two major findings about the mechanisms of multisensory integration for stimuli from the lower limbs and the sense of leg ownership. First, the data show that the first-person visuo-spatial viewpoint enhances the sense of leg ownership and the interference of visual over tactile cues at the lower limbs. This finding further extends the important role of visual top-down factors, here the first-person viewpoint, to the integration of leg-related multisensory stimuli and leg ownership as previously shown for hands (Pavani et al., 2000; Zopf et al., 2010) and the full body (Petkova et al., 2011; Maselli and Slater, 2014). Second, we show that the viewpoint effect on the multisensory integration of stimuli from the lower limbs is stronger when legs are shown, as the CCE magnitude decreased for non-leg control objects.

### Top-down Modulation of the Crossmodal Congruency Effect

The CCE RTs revealed the highest impact of visual distractors when the virtual legs were presented from the first-person viewpoint, indicating a stronger degree of visuo-tactile interference compared to third-person viewpoints and compared to the wooden blocks seen from the first-person viewpoint. Thus, the CCE, based on dominance of task-irrelevant visual stimuli over tactile stimuli, decreased when the legs were viewed in the third-person visuo-spatial viewpoint (also not differing in magnitude from the condition in which the blocks were presented in the third-person viewpoint). Similarly, the effect of visuo-spatial viewpoint on multisensory integration was also reflected in ERs, which decreased when the legs or objects were presented in the third-person viewpoint. The current findings show that visuo-tactile integration for the legs as quantified by the CCE does not only depend on the bottom-up, temporo-spatial visuo-tactile stimulation (e.g., prior visuo-tactile tapping), but also on two visual top-down factors: a pre-existing internal body representation (i.e., corporeal similarity of the virtual legs) and the visuo-spatial viewpoint.

The performance on the CCE task has been associated with the activity of multimodal neurons in premotor and posterior parietal regions, which respond to stimulation within their tactile receptive fields on the body as well as to visual stimuli appearing within the peripersonal space surrounding the given body part despite positional changes of the body part (Maravita et al., 2003; Macaluso and Maravita, 2010). As such it has been proposed that they process multisensory stimuli in a common body-centered (arm- or leg-centered) reference frame (Rizzolatti et al., 1997; Graziano and Gross, 1998; Graziano et al., 2000; Pouget et al., 2002; Avillac et al., 2005). It has been suggested that such body-part centered multisensory coding of stimuli, implemented in premotor and posterior parietal multimodal neurons do not only map the location of body parts in space (Lloyd et al., 2003), but are also fundamental in body ownership (Maravita et al., 2003; Ehrsson et al., 2004; Makin et al., 2007; Zopf et al., 2010; Blanke, 2012).

In our study, viewing the wooden objects in first-person viewpoint reduced the CCE amplitude as compared to viewing the virtual legs in the same viewpoint. Comparable results were also reported in a study with the full body illusion paradigm (Aspell et al., 2009, 2010), where seeing a body modulated the CCE but not when seeing a body-sized control object. We found that visuo-tactile integration as quantified by the CCE was further decreased when the legs or wooden blocks were presented in the rotated orientation, mimicking a third-person viewpoint (i.e., providing a visual reference frame that did not match the egocentric, somatotopic and proprioceptive) reference frame of the legs, compatible, with weakened visuo-tactile integration due to misalignment (i.e., the coordinates within which the visual distractors were mapped were not aligned with the somatotopic coordinates of the vibro-tactile stimuli). Similar findings have been reported for hands, where the tactile stimuli were remapped to the location of visual distractors superimposed over rubber hands, but only when they were spatially aligned with the subject’s real hands, and not when rotated by 90°, even if this posture is physically possible for the hands but not for the legs (Pavani et al., 2000). Comparable effect of the viewpoint on the RT CCE was also shown for the whole body (Maselli and Slater, 2014). Altogether, our CCE results suggest that the multisensory representation of the lower limbs is susceptible to changes of the visual reference frame, as shown for hands and trunk (Tsakiris and Haggard, 2005; Costantini and Haggard, 2007; Heed and Röder, 2012). The first-person visuo-spatial viewpoint thus contributes to successful integration of leg-related visuo-tactile stimuli.

Contrary to our predictions, the synchrony of visuo-tactile leg stimulation prior to the CCE task did not significantly modulate the CCE score, while it instead increased the subjective sense of body ownership as assessed by the questionnaire (see below). The lack of a synchrony effect shows that the congruent visual and proprioceptive information about appearance and position of the legs alone was sufficient to induce the observed changes in the CCE performance, without requiring additional visuo-tactile synchronous tapping (Pavani et al., 2000; Slater et al., 2010; Maselli and Slater, 2013, 2014; van Elk et al., 2013). Alternatively, as the delivered visual and vibrotactile stimuli in the crossmodal congruency task are temporally synchronized, the task itself might have generated some effects, resembling those of the illusion *per se* and canceling out any condition difference due to prior visuo-tactile tapping. Similar findings on the lack of the visuo-tactile tapping synchrony effect on the CCE have been reported for the rubber hand, where the vibro-tactile and visual stimuli were presented simultaneously (Zopf et al., 2010) and for the full-body illusion, when a temporal delay of 33 ms was used (Aspell et al., 2009). However, when a larger delay of 150 ms (Zopf et al., 2010) or 233 ms (Aspell et al., 2009) was introduced between the visual and vibro-tactile stimuli, the CCE was significantly modulated, also by the synchrony of prior stroking. Thus, simultaneous presentation of visual and vibro-tactile stimuli during the present CCE measurements might have reset any synchrony-specific effect due to previous stimulation (Zopf et al., 2010).

### Multisensory Representation of Upper and Lower Body Parts

The present study can also offer an insight into potential differences between upper and lower extremities in terms of their multisensory representation. Although our results cannot be directly compared to the existing studies due to different methodologies, experimental designs, and because we did not measure hand responses in the present study, some considerations are merited. Thus, our CCE results closely relate to the study of Pavani et al. (2000), which showed that anatomically incongruent posture of the rubber hand (rotated by 90°) reduces the CCE, which was comparable to the level where no rubber hand was presented in that study. However, a series of studies demonstrated that showing a photograph or a contour drawing of a hand during a CCE task resulted in relatively high CCE amplitude even if the presented hand image was rotated by 45 or 180°, or shown orthogonally to the participant’s real hand (Igarashi et al., 2004, 2007). Compared to the results of the present study, where the presentation of lower limbs in a tilted orientation significantly decreased CCE magnitude (but not completely abolished it), these previous findings indicated that the hands’ multisensory representation and its surrounding space might be more plastic than those of the lower extremities. Only few studies directly compared the multisensory representation of feet and hands and showed that the feet representation does not differ from that of the hands as inferred from the temporal order judgment tasks and CCE (Schicke and Röder, 2006; Schicke et al., 2009; van Elk et al., 2013). However, it was shown that anatomical incongruence and crossed posture modulated the CCE for the hands but not for the feet, indicating that visual information might be more strongly integrated with tactile and proprioceptive signals for the hands as compared to the feet (van Elk et al., 2013).

Although differences in the multisensory representation between upper and lower extremities might be assumed due to the fact that normally, hands can be positioned in different orientations and are used for frequent manual actions (providing greater variability of visual and proprioceptive information regarding their location than legs), we cannot conclude based on our leg data whether these differences exist, requiring direct comparisons between upper and lower limbs in sensitivity (or robustness) to various deviations from the habitual first person viewpoint.

### Subjective Experience of Embodiment

At the phenomenological level, all three experimental factors—corporeal similarity, first-person visuo-spatial viewpoint and synchrony of stroking contributed to the illusory sense of ownership over virtual legs and illusory touch.

The present study confirms a large body of data showing that bottom-up as well as top-down signals contribute to the sense of hand and full-body ownership, and is thus in accordance with previous studies using the RHI paradigm, which demonstrated that sense of ownership for a hand emerges from spatiotemporal congruence of visual, tactile and proprioceptive cues as well as pre-existent body representations, including the anatomical resemblance (Tsakiris and Haggard, 2005), rules of general body configuration (Farne et al., 2000; Pavani et al., 2000; Austen et al., 2004; Ehrsson et al., 2004; Tsakiris and Haggard, 2005) and laterality (Tsakiris and Haggard, 2005; Tsakiris, 2010; Tsakiris et al., 2010). The present data conforms to a neurocognitive model of body-ownership based on the rubber hand illusion (Tsakiris, 2010) and extends its validity to the lower extremities. According to that proposal, the experience of illusory ownership is established by first comparing the visual, anatomical and structural characteristics of the viewed object with a pre-existing body model, and secondly, the current postural and anatomical features of own body with those of the viewed object. Then the system compares the reference frames of current synchronous visual and tactile input, and resolves the multisensory conflict by recalibrating the visuo-tactile coordinates into a unique body-centered reference frame, leading to the touch referral and induction of body ownership. As predicted by the model and observed in our data, incongruences in anatomical shape characteristics between own physical legs and what was visually presented (such as wooden blocks), between postural features (such as discrepancy between actual and observed leg posture in the third-person viewpoint) and asynchronous visuo-tactile stimulation reduce the sense of ownership for the virtual legs. Our data, however, cannot inform whether the order of critical comparisons as suggested by the model is correct.

Important for understanding the different components of the sense of body ownership (Longo et al., 2008), the subjective reports in our study show discrepancy between experienced body ownership and illusory touch. Although illusory leg ownership was significantly modulated by the visuo-spatial viewpoint and corporeal similarity, illusory touch (i.e., perceiving the touch on the virtual leg or wooden object) was experienced also when the synchronous visuo-tactile tapping was applied to the wooden blocks, or when it was applied in the non-habitual visuo-spatial viewpoint. Similar findings were also reported before (for example: Lenggenhager et al., 2007; Hohwy and Paton, 2010, study 2). The fact that we observed a modulation of illusory touch without illusory leg ownership (in case of wooden objects or third-person viewpoint) indicates a dissociation of the two phenomena, contradicting arguments that illusory touch (or referral of touch) is a sufficient marker of body ownership (Makin et al., 2008). The dissociation between illusory touch and ownership was also found by a comprehensive principal component analysis of subjective reports on the RHI (Longo et al., 2008) and described in neurological patients with somatoparaphrenia, who deny ownership for their left hand, but nevertheless can feel being touched on the very hand (Aglioti et al., 1996; Bottini et al., 2002). Additional evidence for the dissociation between illusory touch and ownership stems from an ERP study on the RHI (Press et al., 2007), showing that synchronous visual and tactile stimuli enhance early somatosensory SEP components regardless whether the visual stimulus is applied to a life-like virtual hand or non-bodily object, whereas later negative SEP components were reported to increase only with respect to the anatomical resemblance of the viewed object, suggesting temporally distinct contributions of the bottom-up and top-down mechanisms to the RHI. Based on the present findings, the experience of illusory touch mainly depends on the temporal correlation of visuo-tactile stimuli and it is less affected by violations of anatomical and postural congruency, whereas in addition to the visuo-tactile spatiotemporal correlation, top-down effects such as first-person visuo-spatial viewpoint and anatomical resemblance determine the experience of illusory body ownership.

### Comparing CCE Task and Subjective Experience of Ownership

A comparison between the present CCE task results and subjective ratings reveals several differences. First, the synchrony of visuo-tactile stimulation significantly increased the experience of illusory leg ownership in comparison to asynchronous stimulation as assessed by the questionnaire, whereas this difference was absent in the CCE results. As already mentioned earlier in the discussion, the discrepancy might stem from the simultaneous onset of vibro-tactile and visual cues in the CCE task, which might have been a form of synchronous multisensory stimulation directly modulating the leg ownership illusion potentially dominating the effects of prior stroking on the CCE results. Second, we have also observed differential contributions of the two experimental factors (visuo-spatial viewpoint and corporeal similarity) to the multisensory effects CCE and the explicit feelings (questionnaire) related to leg ownership. The magnitude of the CCE was modulated by the interaction between the two factors, i.e., the strongest effect was observed when the legs, and not the objects, were presented in the first person visuo-spatial viewpoint, whereas according to the item ratings, both experimental manipulations, independently of each other, affected the subjective experience of leg ownership.

These discrepancies suggest that both measures capture two related, but not fully overlapping processes. The CCE reflects the processing and integration of multisensory stimuli in peripersonal space. The change in the CCE induced by our experimental manipulation, therefore, at most reflects a modulation in the representation of the space surrounding the body part, which in turn may depend on the way that body part is represented and perceived. Questionnaire data, instead, tap on the subjective feeling related to body experience. Thus the strength of visuo-tactile interactions, as measured by the CCE, cannot be directly equated with the subjective sense of ownership, although changes in the CCE may reflect concurrent changes in body experience (Maravita et al., 2002a,b; Pavani and Castiello, 2004; Aspell et al., 2009; Zopf et al., 2010; Sengul et al., 2012; Maselli and Slater, 2014). Also, the brain activity associated with the integration of visual, tactile and proprioceptive information in the rubber hand illusion has been found in several brain regions, including ventral intraparietal sulcus, premotor cortex, lateral occipital complex, operculum and cerebellum (Ehrsson et al., 2004, 2005; Makin et al., 2007), whereas an increased brain activity associated with the conscious experience of ownership for a rubber hand has been observed only in the ventral premotor area (Ehrsson et al., 2004, 2005) and in the posterior insula (Tsakiris et al., 2007), again compatible with the presence of shared and distinct mechanisms.

Future research may address the open issues and limitations of the current study, and in particular investigate the relationship between multisensory integration and subjective sense of ownership. This could, amongst others, include probing the effects of different temporal delays between distractors and target stimuli on CCE and questionnaire measures. This could also be tested in the presence or absence of prior induction of illusion with visuo-tactile tapping.

## Conclusion

In sum, the present study shows that decreased corporeal similarity and larger divergence from the habitual first-person viewpoint reduce the sense of ownership for lower extremities and lessen the integration of visuo-tactile stimuli. By using the virtual leg illusion the study contributes to the understanding of how the multisensory lower extremities are represented in the brain. Understanding the neural mechanisms and the determinants of conscious experience for hands and legs might have important translational application in patients with the central and peripheral neural damage affecting the functionality and the perception of one’s own body (Ehrsson et al., 2008; Marasco et al., 2011; Lenggenhager et al., 2015). While many data are already available in the case of upper limb representation, knowledge concerning the lower limb is much poorer. Considering the large number of patients with disabilities affecting the lower-limbs, due to spinal cord injury or lower limb amputation, these available hand data on multisensory mechanisms and bodily experience may not be sufficient and need to be extended by leg data. Research on multisensory stimulation paradigms and bodily illusions modulating lower limb ownership might be particular relevant especially for technological devices and new rehabilitation protocols aimed at restoring lower limb functions. Our study on the one hand confirms the importance of top-down signals for leg representations and, on the other hand, proposes a sensitive and easy-to-apply paradigm to measure the extent of body ownership and embodiment specifically for the lower limb.

### Conflict of Interest Statement

The authors declare that the research was conducted in the absence of any commercial or financial relationships that could be construed as a potential conflict of interest.
